# Population demography of an endangered lizard, the Blue Mountains Water Skink

**DOI:** 10.1186/1472-6785-13-4

**Published:** 2013-02-13

**Authors:** Sylvain Dubey, Ulrich Sinsch, Maximilian J Dehling, Maya Chevalley, Richard Shine

**Affiliations:** 1 University of Lausanne, Department of Ecology and Evolution, Biophore Bld, Lausanne, 1015, Switzerland; 2 Universität Koblenz-Landau, IfIN, Department of Biology, Universitätsstr. 1, Koblenz, D-56075, Germany; 3 University of Sydney, School of Biological Sciences A08, Sydney, NSW, 2006, Australia

**Keywords:** Australia, Montane species, Reptile, Skeletochronology

## Abstract

**Background:**

Information on the age structure within populations of an endangered species can facilitate effective management. The Blue Mountains Water Skink (*Eulamprus leuraensis*) is a viviparous scincid lizard that is restricted to < 40 isolated montane swamps in south-eastern Australia. We used skeletochronology of phalanges (corroborated by mark-recapture data) to estimate ages of 222 individuals from 13 populations.

**Results:**

These lizards grow rapidly, from neonatal size (30 mm snout-vent length) to adult size (about 70 mm SVL) within two to three years. Fecundity is low (mean 2.9 offspring per litter) and is affected by maternal body length and age. Offspring quality may decline with maternal age, based upon captive-born neonates (older females gave birth to slower offspring). In contrast to its broadly sympatric (and abundant) congener *E. tympanum*, *E. leuraensis* is short-lived (maximum 6 years, vs 15 years for *E. tympanum*). Litter size and offspring size are similar in the two species, but female *E. leuraensis* reproduce annually whereas many *E. tympanum* produce litters biennially. Thus, a low survival rate (rather than delayed maturation or low annual fecundity) is the key reason why *E. leuraensis* is endangered. Our 13 populations exhibited similar growth rates and population age structures despite substantial variation in elevation, geographic location and swamp size. However, larger populations (based on a genetic estimate of effective population size) contained older lizards, and thus a wider variance in ages.

**Conclusion:**

Our study suggests that low adult survival rates, as well as specialisation on a rare and fragmented habitat type (montane swamps) contribute to the endangered status of the Blue Mountains Water Skink.

## Background

Estimation of the age structure of endangered populations can facilitate their efficient management (e.g. [1-3]). Unfortunately, an animal’s age is difficult to calculate for many species. Mark-recapture analyses provide the most direct information, but require long-term studies, precluding any rapid conservation management plans [1]. An alternative way to estimate an individual’s age is skeletochronology, based on histological analyses of growth marks in the skeleton [4,5]. This technique relies upon seasonal variation in rates of skeletal growth, and thus is especially effective for ectothermic vertebrates that live in highly seasonal environments [6,7].

The Blue Mountains Water Skink (*Eulamprus leuraensis*, Wells & Wellington, 1983) is a medium-sized (total length to 20 cm) viviparous scincid lizard that is restricted to less than forty small montane swamps (typically, *<*2 ha) at 560 to 1,060 m elevation in the Blue Mountains and Newnes Plateau, west of the city of Sydney in south-eastern Australia. This species is classified as “endangered” under the IUCN Red List [8], the Threatened Species Conservation Act [9] and the Environmental Protection and Biodiversity Conservation Act [10]. This lizard species is an ecological specialist, and comprises isolated small populations that are subject to considerable ongoing threats. In addition, the species’ entire distribution (montane swamps; **<**2,000 km^2^**)** is listed as threatened under the Threatened Species Conservation Act [9] due to impacts of nearby urbanisation, (i.e., invasion by weeds, modification of the hydrological system and of bushfire regimes, pollutants and longwall mining).

These threats to population persistence are exacerbated by the low vagility of the lizards. Gene flow between populations is very limited, and animals within each of the two major parts of the species’ distribution (Blue Mountains versus Newnes Plateau) have been isolated from each other for at least a million years [11,12]. In addition, this species has low annual fecundity (one to five neonates per annual litter; [13,14]), further reducing the ability of populations to recover from the effects of episodes of higher-than-usual mortality [15,16].

In the present study, we used phalangeal skeletochronology (supported by field mark-recapture data) to estimate the age structure of 13 populations of Blue Mountains Water Skinks. As well as clarifying basic issues such as growth rates and age at maturity, we took advantage of data from our earlier studies to explore the effects of maternal age on reproductive output and progeny quality (based on data from Dubey *et al.*, [13] and Dubey & Shine, [14]) and to compare population age structure to various site-specific parameters (including indices of genetic diversity from Dubey & Shine, [11,12]).

## Results

**Figure 1:**
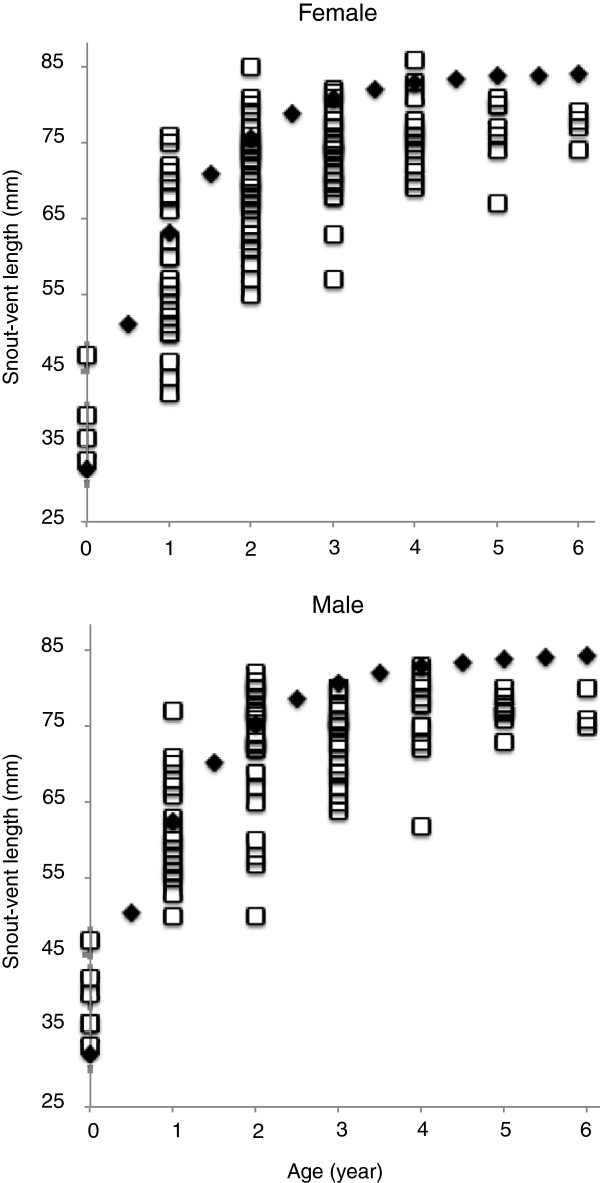
**The relationship between lizard age and body size in *Eulamprus leuraensis *of both sexes, based on skeletochronology of 220 lizards, compared to results from the von Bertalanffy growth model applied to data from lizards captured and then recaptured in the following year. **The black squares show the von Bertalanffy curve, and the grey dots show data for individual lizards.

Our mark-recapture data show that Blue Mountains Water Skinks exhibited rapid growth in the first two years after birth (Figure 1). Our skeletochronological estimates of age (= n LAGs – 1) agree well with those predicted (based on body size) by the Von Bertalanffy function fitted to our mark-recapture data (Figure 1). Born at approximately 30 mm snout-vent length and weighing 0.7 g [14], these lizards attained maturation at SVLs of about 70 mm SVL and 7 g (this study; [13]) within two to three years (Figure 1). Growth slowed thereafter, eliminating any correlation between age and body size in lizards that were more than three years of age (Figure 1).

**Figure 2:**
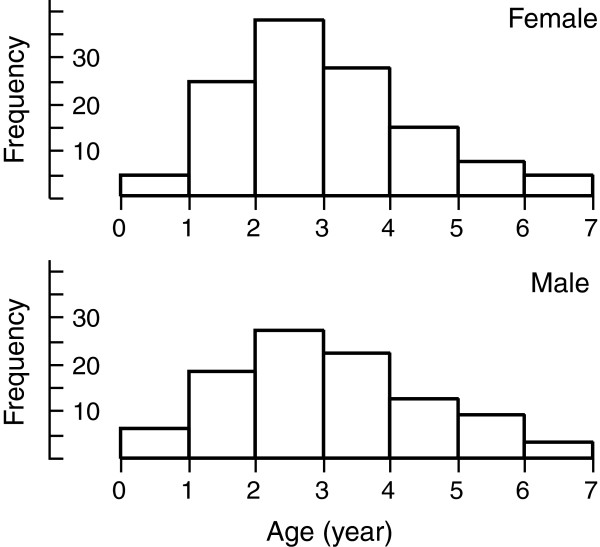
**The overall age structure of male and female Blue Mountains Water Skinks, *Eulamprus leuraensis *(data combined from all populations).**

Our age estimates suggest that the oldest males and females among our sample of 222 lizards were only six years old. About half (49%) of the adult lizards (roughly two or more years in age) were two years old, and only 23% were more than three years old (see Figure 2). Most of the lizards in our study population may live to reproduce only once or twice before dying. The mean age of males that sired offspring (based on paternity analyses from Dubey *et al.*[13]) was 2.88 years (N = 18; varying from one to five) and the mean age of adult males (i.e., > 52 mm SVL) for which juveniles were not assigned was 2.25 years (N = 16; from two to six). The mean age of females that produced litters was 3.00 years (N = 38; from two to six years old) whereas three adult (SVL > 66 mm) but nonreproductive females averaged 1.33 years of age (from one to two). The probability of reproducing increased with age after maturation in females (F_1,40_ = 7.40, P < 0.01) but not males (F_1,33_ =2.59, P = 0.11).

Our multiple linear regression including the age and size (SVL) of gravid females as explanatory variables and the litter size as the response variable, revealed a significant effect of a female’s body size (F_1,39_ = 28.99, P < 0.0001) and of the interaction term between age and body size (F_1,39_ = 6.16, P = 0.018) on her litter size, as well as a marginally significant effect of her age (F_1,39_ = 3.62, P = 0.065). Older and larger females produced larger litters.

We also found a significant effect of these parameters on mean offspring size (SVL), with larger females producing larger neonates and older females smaller neonates (age: F_1,34_ = 8.79, P = 0.006; size: F_1,34_ = 4.63, P = 0.039; interaction: F_1,34_ = 5.59, P < 0.025). Similarly, larger females produced heavier neonates whereas older females gave birth to lighter neonates (age: F_1,34_ = 4.65, P = 0.039; size: F_1,34_ = 1.01, P = 0.32; interaction: F_1,34_ = 6.24, P = 0.018).

**Figure 3:**
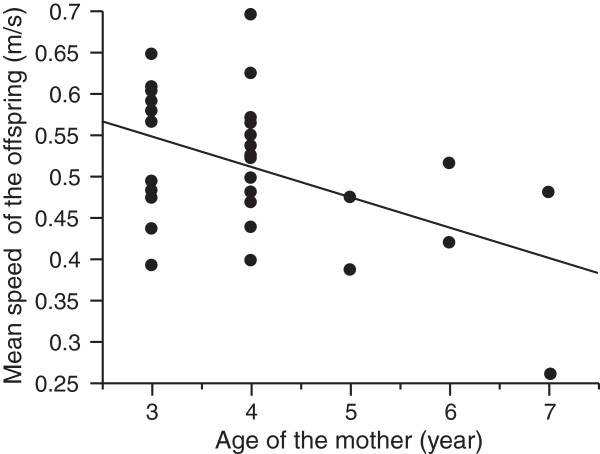
Relationships between the age of female lizards and the mean sprint speed of their offspring.

Interestingly, our performance tests on offspring born in captivity showed that the progeny of older females were also slower (analysis based on mean speeds per litter, age: F_1,30_ = 8,18; P = 0.0078; size: F_1,30_ = 0.45, P = 0.5; interaction: F_1,30_ = 0.01, P = 0.9; Figure 3). Finally, we found no significant relationship between a mother’s age and her snout-vent length (F_1,36_ = 1.13, P = 0.30).

**Figure 4:**
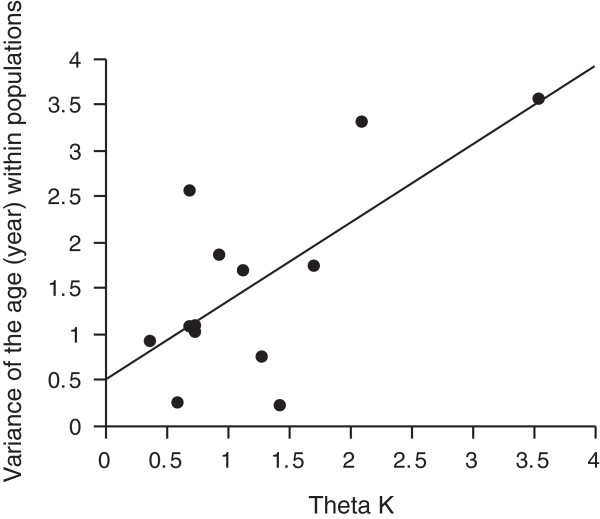
Relationships between the genetically-based population parameter Theta k and the variance in lizard ages (years) within 13 populations of the Blue Mountains Water Skink.

Blue Mountains Water Skinks are surprisingly short-lived. The mean age of individuals within populations varied from 1.67 (BH5) to 3.75 (BH3) years (Table 1), and differed significantly among populations (one-way ANOVA: F_12,206_ = 3.19, P = 0.0004). The oldest individuals were six years old (Figure 2). We found no significant relationship between the mean age of individuals within populations and the elevation (F_1,12_ = 1.77; P = 0.21), longitude (F_1,12_ = 1.32; P = 0.27) or latitude (F_1,12_ = 2.71; P = 0.13) of sites of collection, or the size of the swamps (F_1,12_ = 0.14; P = 0.72). However, both the mean age of individuals, and the variance in ages within a population, were higher in larger populations (using Theta k as our measure of effective population size; Theta k *versus* mean age: F_1,12_ = 4.97; P = 0.048; *versus* variance in ages: F_1,12_ = 9.73; P = 0.0097; Figure 4).

**Table 1:** **Mean age (with variance in parentheses) of *Eulamprus leuraensis *in our study populations**

**Population**	**Mean age**	
BH3	4.75	(3.31)
BH4	3.33	(1.08)
BH5	2.67	(0.22)
MH4	3.5	(0.25)
MRP1	3.5	(0.75)
WFL	4.2	(3.56)
KT1	3.44	(1.02)
WF7	2.95	(1.09)
WF5	2.71	(0.92)
WF1	3.8	(2.56)
XFC1	3.79	(1.86)
NP4	3.86	(1.74)
PNP1	4.25	(1.69)

## Discussion

Blue Mountains Water Skinks grow fast and die young. The largest cohort of adult females comprised those in their first reproductive year post-maturation. Older females were rare, and (because they ceased growing) did not produce larger litters than their younger counterparts. Also, the offspring of older females exhibited reduced locomotor performance. In combination, these traits suggest that the reproductive capacity of a population of Blue Mountains Water Skinks rests primarily upon the output of newly-matured animals. This result provides a striking contrast to the demography of a congeneric water skink that is also found at relatively high elevations (and indeed, occurs in the eucalypt woodland of the Blue Mountains). The highland water skink *E. tympanum* is an abundant and widely-distributed taxon that has been the subject of detailed ecological studies, including skeletochronological work ([17-22]). Tilley [17] reported females living up to at least 15 years of age in a Victorian population, and Blomberg & Shine [22] concluded that adults lived more than 10 years in a NSW population. Mean ages of reproductive adults within *E. tympanum* populations were between four and seven years (in low and high elevation sites, respectively), compared to three to four years in the high-elevation *E. leuraensis* (present study). Offspring mass and litter size are similar in the two species. However, the proportion of females reproducing is higher in *E. leuraensis* than in *E. tympanum* (91% versus 30 to 60%, respectively; [13,19]).

In many lizard and snake species, reproductive output increases strongly with maternal age, primarily because continued growth translates into larger females, that reproduce more often and produce more offspring when they do so (e.g., [23]). Blue Mountains Water Skinks do not exhibit such an increase. Instead, reproductive frequency is as high from the outset (as soon as females mature) as thereafter, because body size (the main determinant of litter size) does not increase substantially during adult life. Indeed, offspring quality (and thus, the benefits to maternal fitness) may decline in older females, based on the reduced locomotor capacities and body sizes of their offspring. Given a mean litter size of 2.9 [13,14], the average lifetime production of neonates per female (even if she reaches six years of age) is less than 15. The short adult lifespan, coupled with the small litter size, must reduce the rate that a population can recover after its numbers have been reduced by events such as bushfires, heavy predation, or prolonged drought. Clearly, the survival rate of the newborns will have a major impact on the rate of population recovery.

The age structure of our populations suggests that many of the lizards are semelparous: that is, they reproduce in only a single year before dying. Like most cool-climate viviparous squamates, Blue Mountains Water Skinks produce only a single litter each year (although females in tropical populations of the congeneric *E. quoyii* can produce two litters per year: Schwarzkopf, unpublished). Although most lizards and snakes are iteroparous, semelparity is not uncommon, especially in harsh habitats such as hot and dry areas as well as at high elevations (e.g. [24-28]).

Although the 13 populations that we sampled spanned a broad range in terms of abiotic factors such as elevation, geographic location and swamp size, none of these factors had any detectable effect on the age structure of lizard populations. However, a genetic estimate of the effective population size (Theta k; based on the study of Dubey & Shine, [15]) was positively correlated with mean age and with intrapopulation variation in age. This pattern suggests that areas that confer higher survival of individual lizards (e.g., due to low predation rates or high food availability) thereby result in larger effective population sizes. Intuition suggests that larger swamps should contain more lizards, but our analyses show no such pattern (i.e., neither Theta k nor age structure were correlated with swamp size). Habitat quality may be more important than quantity. A low Theta k value also reflects low genetic diversity, which could directly reduce survival rates via inbreeding effects [29,30]. Future conservation efforts could usefully explore the biotic and abiotic factors that render swamps more or less favorable to Blue Mountains Water Skinks.

Our skeletochronological data also identify a puzzling feature of this endangered lizard’s life history: its low adult survival rate. At a proximate level, this low survival rate must contribute to the precarious conservation status of the Blue Mountains Water Skink. If that low survival rate was driven by extrinsic factors such as local predation pressure or fire history, we would have expected to see at least some populations with much older animals. Thus, our data suggest that either the entire range of this species was affected by some major catastrophe about seven years prior to our study, or else low adult survival rates are a consistent feature of the biology of this species. We know of no obvious historical catastrophe that could explain the truncation of age structures across the entire range of the species. Thus, the most likely scenario is that, unlike its congeners such as *E. tympanum*, the Blue Mountains Water Skink is a relatively short-lived species.

## Conclusions

The analysis above adds a demographic factor to the traits already identified as contributing to the endangerment of *E. leuraensis*. These lizards are habitat specialists, dependent upon a relatively scarce and highly fragmented set of highland swamps [11,31,32]. Their low vagility reduces gene flow among swamps, and retards recolonisation of any swamps where local populations have been extirpated [12,13]. They live in an area that abuts Australia’s largest city, and hence are exposed to myriad threats caused by the proximity of humans [11,31,32]. Their dependence on moist habitats at high elevations means that they are at a high risk of habitat degradation due to climate change (southeastern Australia is predicted to become hotter and drier: [33]). Lastly, they exhibit the unfortunate combination of low reproductive output and low rates of adult survival. In combination, these characteristics render the Blue Mountains Water Skink highly vulnerable to any additional pressures (e.g., [13,34,35]), and suggest that the future conservation and management of this species will require close scrutiny and perhaps, active intervention to maintain suitable habitat conditions.

## Methods

### Sampling procedure

**Figure 5:**
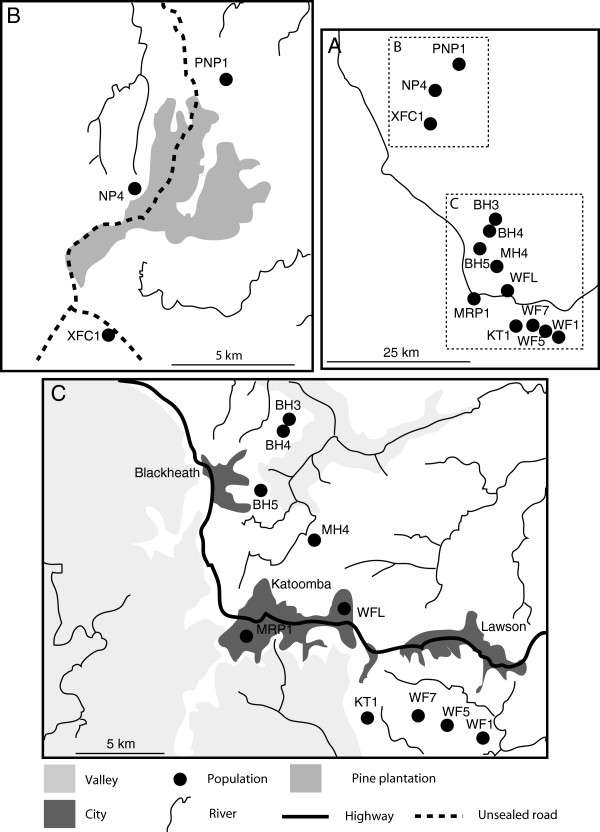
**Distribution of the 13 populations of Blue Mountains Water Skinks (*Eulamprus leuraensis*) analysed in the present study (A: entire range; B: Newnes Plateau; C: Blue Mountains).**

In the course of our genetic studies [12-14], we collected toe-clips from 222 individuals in 13 different sites from mid-spring to mid-summer 2008–2009 and spanning virtually the entire known range of the species (Figure 5). We recorded the sex, total length, snout-vent length and tail length (to the nearest mm), and mass (to the nearest 100 mg) before toe-clipping the animal and releasing it at its site of capture. The phalange samples were stored in 90% ethanol at room temperature for skeletochronological age assessment.

The toe-clips also provided an opportunity to mark individuals with a unique toe-clip code and hence to assess growth rates (and thus, ages relative to body size) by calculating growth increments for the 27 individuals that we recaptured in successive years. We used the von Bertalanffy growth model [36,37] to estimate the relationship between size and age for both males and females. We fitted mark-recapture data to these models using the non-linear least-squares regression procedure in JMP 8.0.1 [37-39]. The parameters k (intrinsic growth rate) and a (asymptotic length) were seeded with initial best guesses.

### Skeletochronological age estimation

Laboratory procedures for processing humeri and the second phalanx of the toes followed the standard methods of skeletochronology for amphibians and lizards [4,40]: (1) decalcification of bones (5% formic acid, 1 h), (2) fixation in Bouin´s solution (at least 1 h), (3) Historesin™ (JUNG) embedding, (4) cross sectioning of the diaphysis at 10 μm using a JUNG RM2055 rotation microtome, (5) staining with 0.05% cresylviolet (5-10 min), (6) light microscopic examination using an Olympus BX 50 with ocular micrometer. The number of lines of arrested growth (LAG) was counted in the periosteal bone of those diaphysis sections in which the size of the medullar cavity was at its minimum and that of bone at its maximum. The number of usable diaphysis sections per individual varied between 2 and 16. All were considered for LAG counting and two of the authors independently obtained age estimates for each individual. Endosteal bone was present in all individuals, but the first LAG always remained visible (maximum resorption: ca. 30% of the complete line).

**Figure 6:**
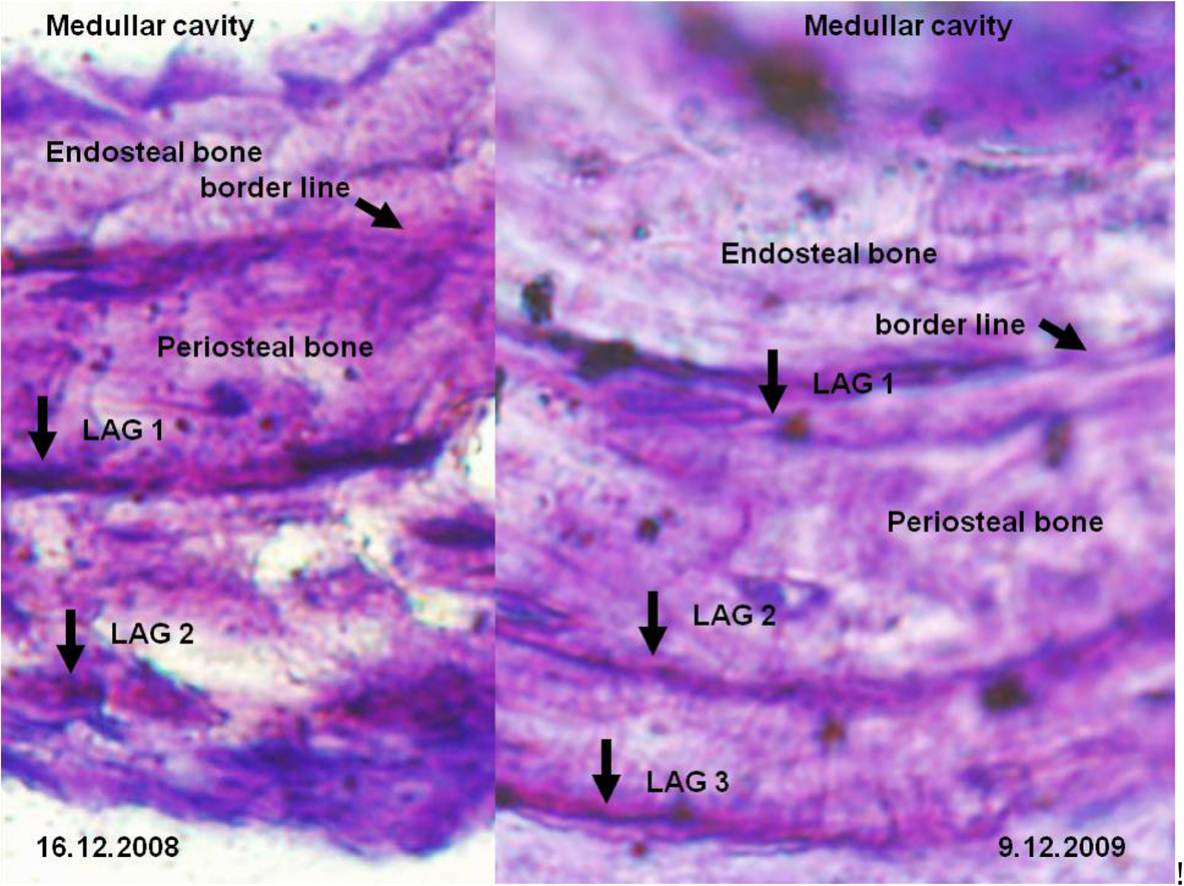
**Histological section of the second phalanx of a female Blue Mountains Water Skink collected in two successive years at locality KT1. **When first captured on 16 December 2008, this animal measured 71 mm snout-vent length and weighed 7.9 g; when recaptured on 9 December 2009 it measured 80 mm SVL and weighed 11.5 g.

The validity of LAGs as age estimators was supported by our observations that (a) in four recaptured individuals for which we obtained successive samples one year apart, the number of LAGs had increased by 1 in each case (see Figure 6 for an example); (b) the location of the LAG relative to the growing edge of the bone shifted seasonally, in a predictable fashion (in spring, the LAG was near the outer edge of the bone; in autumn, the LAG was separated from the bone edge by a large growth zone); and (3) all captive-born neonates had only a single LAG. Consequently, the age of an individual corresponds to the number of LAGs minus 1.

### Statistical analyses

Statistical analyses were conducted using JMP 8.0 [39]. Linear regressions were performed to compare the mean age of individuals within populations to longitude, latitude, elevation, swamp size and an estimate of the effective population size based on genetic analyses from Dubey & Shine ([11]; Theta k, estimated from the infinite-allele equilibrium relationship [41] between the sample size [n], the expected number of alleles [k], and Theta [population parameter; Theta = 2Mu where M is equal to 2 N for diploid populations of size N, and u is the overall mutation rate at the haplotype level]; [42]).

We also performed multiple linear regressions between the age and size (SVL) of gravid females and their reproductive output (litter size and offspring size), including hatchling locomotor performance (sprint speed: see Dubey & Shine, [15] for methods). We omitted variables (incubation treatments, litter sizes) that did not affect locomotor speeds in this system [15]. Mean values were calculated for each litter to avoid pseudoreplication. Data on reproductive variables were obtained from Dubey & Shine [15] and Dubey *et al.*[14]. Based on these data and paternity analyses from Dubey *et al.*[14], we also estimated the mean age of males that were known to have sired offspring.

## Competing interests

The authors declare that they have no competing interests.

## Authors’ contributions

SD, MC, US, JMD and RS contributed with the conceptual development of the work and the writing of the manuscript. SD, JMD, and US carried out the analyses and SD and MC performed the fieldwork. All authors read and approved the final version of the manuscript.
